# Editors’ Book Table

**Published:** 1873-02

**Authors:** 


### Editors' Book Table.

[NOTE. — All works reviewed in the columns of the CHICAGO MEDICAL JOURNAL may be found in the extensive stock of W. B. KEEN, COOKE & Co., whose catalogue of Medical Books will be sent to any address upon request.]

BOOKS RECEIVED.

*The Pharmacopœia of the United States of America.* Fifth Decennial Revision. By authority of the National Convention for Revising the Pharmacopœia, held at Washington, A. D. 1870. Philadelphia: J. B. Lippincott & Co. 1873. Pp. 383. Cloth, $1.75.

The present revision of the Pharmacopœia is more complete and extensive than we had supposed would be the case, and both physicians and druggists should procure this book and take appropriate note of changes. Twenty-four medicines have been added to the primary list, and three to the secondary. One substance is dropped from the primary and four from the secondary list. Eighty-two preparations have been added and seven omitted. As new classes we find *Chartae* (including Cantharides and Mustard paper), *Glycerita*, *Suppositoria* and *Succi*, which latter explain themselves. The new chemical nomenclature is adopted only to a limited extent, and numerous minor changes made necessary by advances or changes in chemistry have been introduced. The committee of ten have well performed their duty, and the publishers have brought out the book in their well-known neat and attractive style.

*The Practice of Surgery.* By THOMAS BRYANT, F.R.C.S., Surgeon to Guy's Hospital. With 507 illustrations. (8vo. Pp. 984. Extra cloth, $6.25; Raised bands, leather, $7.25.) Philadelphia: Henry C. Lea. 1873.

The vast experience and distinguished reputation of the author of this book, led us to anticipate a valuable, useful and practical work from his hands, and in this we are not disappointed in looking it over. The accumulated stores within the walls of "Guy's" having been placed freely at the disposal of the author, have enabled him to write *ex cathedra* with the authority of unusually large experience. Although briefly entitled "The Practice of Surgery," it really embraces "large discourse" upon guiding principles. Most of the numerous illustrations are original, prepared expressly for this book. Taking it together, we commend it as a capital contribution to surgical literature, largely worthy of being added to the libraries of our readers.

*Wohler's Outlines of Organic Chemistry.* By Rudolph Fittig, Ph. D., Nat. Sc. D., Professor of Chemistry in the University of Tubingen. Translated from the Eighth German Edition with additions, by Ira Remsen, M.D., Ph. D., Professor of Chemistry and Physics in Williams College, Mass. Philadelphia: Henry C. Lea. 1873. Royal 12mo. Pp. 530. Extra cloth, $3.00.

The number of editions of this work in Germany, attest the high repute it has gained as the leading text book and standard authority. The American edition receives the express approbation of the author, and in it will be found numerous additions and alterations, bringing it up to the latest date. As a work of reference it is invaluable, and by the forethought of the editor it is rendered more than usually so by the free use of large full-faced letters for each prominent article treated of.

*Diseases of the Ovaries: their Diagnosis and Treatment.* By T. Spencer Wells, Fellow and Member of the Royal College of Surgeons, England, Surgeon in ordinary to the Queen's Household, etc., etc., etc. New York: D. Appleton & Company, 549 & 551 Broadway. 1873. 8vo. Pp. 478.

This volume is issued simultaneously in London and New York, and may be considered as the very latest, as it is by far the most satisfactory, if not the best, exposition of the subject. It is clear, concise, thoroughly scientific, and yet practical. We advise every practitioner who has a case of ovarian disease under observation to buy and read this book. Incidentally we notice that Mr. Wells prefers as an anæsthetic chloro-methyl, or, as it is more commonly termed, the bichloride of methylene.

*Parturition Without Pain:* A Code of Directions for Escaping from the Primal Curse. Edited by M. L. Holbrook, M.D., Editor of the "Herald of Health." Third Edition, Enlarged. New York: Wood & Holbrook. 1872. 12mo. Pp. 147.

*Surgical Diseases of Infants and Children.* By M. P. GUERSANT, Honorary Surgeon of the Hopital des Enfants Malades, Paris; Honorary Member of the Societe de Chirurgie, etc. Translated from the French by RICHARD J. DUNGLISON, M.D. Philadelphia: Henry C. Lea. 1873. 8vo. Pp. 354. $2.50.

Briefly, the results of twenty years service in the great Children's Hospital of Paris.

*Obstetric Aphorisms:* For the use of Students Commencing Midwifery Practice. By JOSEPH GRIFFITHS SWAYNE, M.D., Physician Accoucheur to the Bristol General Hospital, and Lecturer on Obstetric Medicine at the Bristol Medical School. Second American from the Fifth Revised English Edition, with Additions. By EDWARD R. HUTCHINS, M.D. Philadelphia: Henry C. Lea. 1873. 12mo. Pp. 189.

This little manual has secured, as we prophesied on its first appearance, deserved popularity.

PAMPHLETS, ETC.

*Fœticide or Criminal Abortion:* A Lecture Introductory to the Course on Obstetrics and Diseases of Women and Children, University of Pennsylvania. By HUGH L. HODGE, M.D. Fourth Edition. Philadelphia: Lindsay & Blakiston. 1872. Paper, 30 cts.; Cloth, 50 cts.

Much good may be done by the further circulation of this lecture.

*Braithwaite's Restrospect of Practical Medicine and Surgery.* Part LXVI. January, 1873. Uniform American Edition. New York: W. A. Townshend, Publisher. $2.50 a year, in advance, Postage Prepaid. Half Yearly Parts, $1.50.

*The Journal of Anatomy and Physiology.* Conducted by G. M. HUMPHREY, M.D., F.R.S., Prof., etc., and by WM. TURNER, M.B., Prof., etc. Second Series. No. XI. November, 1872. Macmillan & Co., Cambridge, London and New York. 8vo. Paper. Pp. 200; and seven pages of lithograph plates. $1.75.

*Transactions of the Wisconsin State Medical Society.* 1872. With the Constitution, By-Laws, and List of Members. Pp. 168.

*Transactions of the New Hampshire Medical Society.* Eighty-Second Anniversary. Held at Concord, June 11th and 12th, 1872. Pp. 96.

*Transactions of the Medical Society of the State of West Virginia*, instituted April 10th, 1867. Pp. 100.

*Chronic Urethral Discharge.* By C. H. MASTIN, M.D.; Mobile, Ala. Read before the Alabama Medical Association. 1872. Pp. 32.

*The Natural Cure of Disease.* Synopsis of a Lecture by Prof. SAMUEL G. ARMOR, M.D.

